# Genome Sequence of *Bacillus safensis* CFA06, Isolated from Biodegraded Petroleum in Brazil

**DOI:** 10.1128/genomeA.00642-14

**Published:** 2014-07-24

**Authors:** Prianda R. Laborda, Francine S. A. Fonseca, Célio F. F. Angolini, Valéria M. Oliveira, Anete P. Souza, Anita J. Marsaioli

**Affiliations:** a Centro de Biologia Molecular e Engenharia Genética (CBMEG), Universidade Estadual de Campinas (Unicamp), Campinas, São Paulo, Brazil; b Instituto de Química, Universidade Estadual de Campinas (Unicamp), Campinas, São Paulo, Brazil; c Centro Pluridisciplinar de Pesquisas Químicas, Biológicas e Agrícolas (CPQBA), Universidade Estadual de Campinas (Unicamp), Campinas, São Paulo, Brazil

## Abstract

*Bacillus safensis* is a microorganism recognized for its biotechnological and industrial potential due to its interesting enzymatic portfolio. Here, as a means of gathering information about the importance of this species in oil biodegradation, we report a draft genome sequence of a strain isolated from petroleum.

## GENOME ANNOUNCEMENT

*Bacillus safensis* is a Gram-positive rod-shaped free-living spore-forming mesophilic bacterium originally isolated from a National Aeronautics and Space Administration (NASA) spacecraft assembly facility (1). The presence of the species has also been reported in desert soil (2), sweet meat whey (3), root tubers (4), and rhizosphere (5). The search for potentially biodegrading microorganisms revealed that this species can also be found in petroleum samples. Since then, ongoing studies are demonstrating its environmental relevance in biocatalysis and bioremediation, and they point to the importance of a genome sequence to complement biological and chemical findings. The first *B. safensis* genome was announced in 2013 and regarded a rhizosphere sample from a saline desert in India (5). In this paper, we present the genome sequence of *B. safensis* strain CFA06, recovered from biodegraded oil samples from the Pintassilgo Field at the Potiguar Basin, in the state of Rio Grande do Norte, Brazil.

A genomic library for *B. safensis* strain CFA06 was constructed according to the recommendations of the TruSeq DNA sample preparation kit (Illumina, Inc.), beginning with 1 µg of sheared DNA. The shotgun sequencing was conducted using one flowcell lane (clusterized with 10 pM) of a Genome Analyzer IIx (Illumina, Inc.) and paired reads of 2 × 72 bases. The CLC Genomics Workbench version 4.9 (CLC bio) was used for read quality control (minimum read *Q* score, 30; maximum ambiguous nucleotides allowed, 2; minimum read length, 70 bases) and *de novo* assembly into contigs (minimum contig length, 450 bp; *k*-mer, 41). Further genomic analyses, such as sequence similarity searching and annotation, were conducted by the Integrated Microbial Genome (IMG) Annotation Pipeline (6).

The whole-genome sequencing of *B. safensis* CFA06 generated >6.8 Gb of data in 94 million paired reads. More than 91% of the reads passed quality control. The *de novo* assembly resulted in 65 contigs, with an average length of 58,020 bp (ranging from 495 to 294,018 bp) and an *N*_50_ contig length of 103,586 bp. The estimated genome size is 3,771,318 bp, with 41.47% G+C content. Approximately 70% of the nucleotide positions had coverage ranging from 1,000 to 2,000×. A total of 3,781 protein-coding genes were predicted, 71% of which had a predicted function. Of the 43 RNA genes identified, 3 are rRNAs and 12 are tRNAs. Twenty-nine percent of the predicted genes (*n* = 1,124) are connected to KEGG pathways.

### Nucleotide sequence accession numbers.

This whole-genome shotgun project has been deposited at DDBJ/EMBL/GenBank under the accession no. JNBO00000000. The version described in this paper is version JNBO01000000. The *B. safensis* genome sequences have also been deposited at the IMG Database under the project ID Gi23929.
